# Deoxynivalenol Exposure Suppresses Adipogenesis by Inhibiting the Expression of Peroxisome Proliferator-Activated Receptor Gamma 2 (PPARγ2) in 3T3-L1 Cells

**DOI:** 10.3390/ijms21176300

**Published:** 2020-08-31

**Authors:** Yurong Zhao, Shulin Tang, Ruqin Lin, Ting Zheng, Danyang Li, Xiaoxuan Chen, Jiahui Zhu, Jikai Wen, Yiqun Deng

**Affiliations:** 1 Guangdong Provincial Key Laboratory of Protein Function and Regulation in Agricultural Organisms, College of Life Sciences, South China Agricultural University, Guangzhou 510642, China; 20171003017@stu.scau.edu.cn (Y.Z.); shulintang@scau.edu.cn (S.T.); linruqin@stu.scau.edu.cn (R.L.); zhengting@stu.scau.edu.cn (T.Z.); lidanyang@stu.scau.edu.cn (D.L.); xuanzi@scau.edu.cn (X.C.); jhz@stu.scau.edu.cn (J.Z.); 2 Key Laboratory of Zoonosis of Ministry of Agriculture and Rural Affairs, South China Agricultural University, Guangzhou 510642, China

**Keywords:** deoxynivalenol, peroxisome proliferator-activated receptor gamma 2, adipogenesis, toxicology, histone modifications

## Abstract

Deoxynivalenol (DON)—a type B trichothecene mycotoxin, mainly produced by the secondary metabolism of *Fusarium*—has toxic effects on animals and humans. Although DON’s toxicity in many organs including the adrenal glands, thymus, stomach, spleen, and colon has been addressed, its effects on adipocytes have not been investigated. In this study, 3T3-L1 cells were chosen as the cell model and treated with less toxic doses of DON (100 ng/mL) for 7 days. An inhibition of adipogenesis and decrease in triglycerides (TGs) were observed. DON exposure significantly downregulated the expression of PPARγ2 and C/EBPα, along with that of other adipogenic marker genes in 3T3-L1 cells and BALB/c mice. The anti-adipogenesis effect of DON and the downregulation of the expression of adipogenic marker genes were effectively reversed by PPARγ2 overexpression. The repression of PPARγ2′s expression is the pivotal event during DON exposure regarding adipogenesis. DON exposure specifically decreased the di-/trimethylation levels of Histone 3 at lysine 4 in 3T3-L1 cells, therefore weakening the enrichment of H3K4me2 and H3K4me3 at the *Pparγ2* promoter and suppressing its expression. Conclusively, DON exposure inhibited PPARγ2 expression via decreasing H3K4 methylation, downregulated the expression of PPARγ2-regulated adipogenic marker genes, and consequently suppressed the intermediate and late stages of adipogenesis. Our results broaden the current understanding of DON’s toxic effects and provide a reference for addressing the toxicological mechanism of DON’s interference with lipid homeostasis.

## 1. Introduction

Deoxynivalenol (DON), a type B trichothecene mycotoxin, is one of the most polluting mycotoxins, and is mainly produced by *Fusarium* [1,2]. DON, also known as “vomitoxin”, causes vomiting in swine [3]. DON has a negative impact on animal health, especially causing gastrointestinal disorders in monogastric animals [4]. Acute exposure to DON leads to vomiting and diarrhea, and is associated with a loss of appetite, while long-term exposure leads to decreased weight gain, intestinal toxicity, cell-mediated immune dysregulation, neurological disorders, lipid peroxidation, and high sensitivity towards disease [5,6,7,8]. The decrease in weight gain caused by DON still remains to be clarified. It is reasonable to consider that a lower intake of food in DON-exposed animal individuals would result in decreased weight gain due to diarrhea and loss of appetite, but it would be interesting to understand whether DON administration could directly inhibit adipogenesis.

Adipogenesis, the differentiation of preadipocytes, is a multi-step process involving the activation of transcription factors and the upregulation of adipocyte-specific genes, which lead to their differentiation to mature adipocytes [9,10,11,12]. Adipogenesis is mainly controlled by two protein families, CCAAT/enhancer-binding proteins (C/EBPs) and peroxisome proliferator-activated receptors (PPARs) [13,14,15,16]. Among these, C/EBPα and PPARγ function as key transcription factors, activating the expressions of a series of adipocyte-specific genes during terminal adipocyte differentiation.

C/EBP proteins are a family of basic region leucine zipper (bZIP) transcription factors that includes six members with related sequences and functions (α, β, γ, δ, ε, and ζ) [17]. C/EBPα is required for the proper control of adipogenesis, glucose metabolism, granulocytic differentiation, and lung development [18]. C/EBPα is induced during late adipogenesis and is most abundant in mature adipocytes, mediating growth arrest and terminal differentiation in concert with PPARγ [19].

PPARs are identified as members of the nuclear receptor superfamily of transcription factors, consisting of three isotypes—PPARα, PPARβ/δ, and PPARγ—distributed in different tissues. PPARγ have two isoforms differing at the amino terminal extension with an extra thirty amino acids in PPARγ2 but not in PPARγ1. PPARγ1 is expressed in multiple tissues, but PPARγ2 is mainly expressed in adipose tissue and strongly upregulated during adipogenesis, suggesting a specific role in adipogenesis. Exogenous PPARγ2 but not PPARγ1 reactivates adipogenesis in PPARγ-knockdown cells [20,21,22,23]. Recent studies have shown that histone modifications are important in regulating the expression of PPARγ, mainly via methylation and acetylation at different amino acids of Histone 3—including H3K4me2/3, H3K9ac, and H3K27ac—associated with transcriptional initiation, “open” chromatin, and cis-regulator activity in the *Pparγ* promoter locus [24,25]. The activity of PPARγ is also regulated by its interaction with other nuclear proteins such as the co-activators [26]. These protein interactions are involved in the expression regulation of adipocyte genes, including fatty acid synthesis acetyl-CoA carboxylase alpha (ACACA), fatty acid synthase (FASN), fatty acid binding protein 4 (FABP4), and fatty acid translocase (CD36), which participate in different adipogenic functions, such as fatty acid synthesis, fatty acid transport, and energy metabolism [27]. ACACA catalyzes the carboxylation of acetyl CoA to form malonyl-CoA, the first committed step in the synthesis of long-chain fatty acids [28]. FASN is a multifunctional enzyme involved in lipogenesis, the synthesis of long-chain fatty acids from acetyl-CoA, and promoting the cytoplasmic storage of triglycerides [11]. FABP4, originally named adipocyte protein 2 [29], is the first identified PPARγ2 target gene and functions in fatty acid transport, facilitating the cellular uptake of long-chain fatty acids for metabolic processing or storage [30]. CD36 was reported to play a significant role in adipocyte cholesterol and adipogenesis metabolism [31,32].

In the present study, the effects of DON exposure on adipogenesis were evaluated for the first time. Our results demonstrated that DON inhibited the expression of adipogenic marker genes, thereby suppressing adipogenesis both in vitro and in vivo. PPARγ2 overexpression rescued the inhibitory effect of DON on lipid accumulation and the expression of adipogenesis marker genes in 3T3-L1 cells. Furthermore, we revealed that it repressed PPARγ2 expression by reducing H3K4me2 and H3K4me3 enrichment at the promoter of *Pparγ2*. Our findings suggested that DON suppressed adipogenesis by inhibiting PPARγ2 expression. The elucidation of its toxicological actions in adipogenesis will provide a theoretical reference for the subsequent exploration of its effects on lipid homeostasis and new insights into its suppression of weight gain.

## 2. Results

### 2.1. DON Suppresses Intracellular Lipid Accumulation in 3T3-L1 Cells

**Figure 1:**
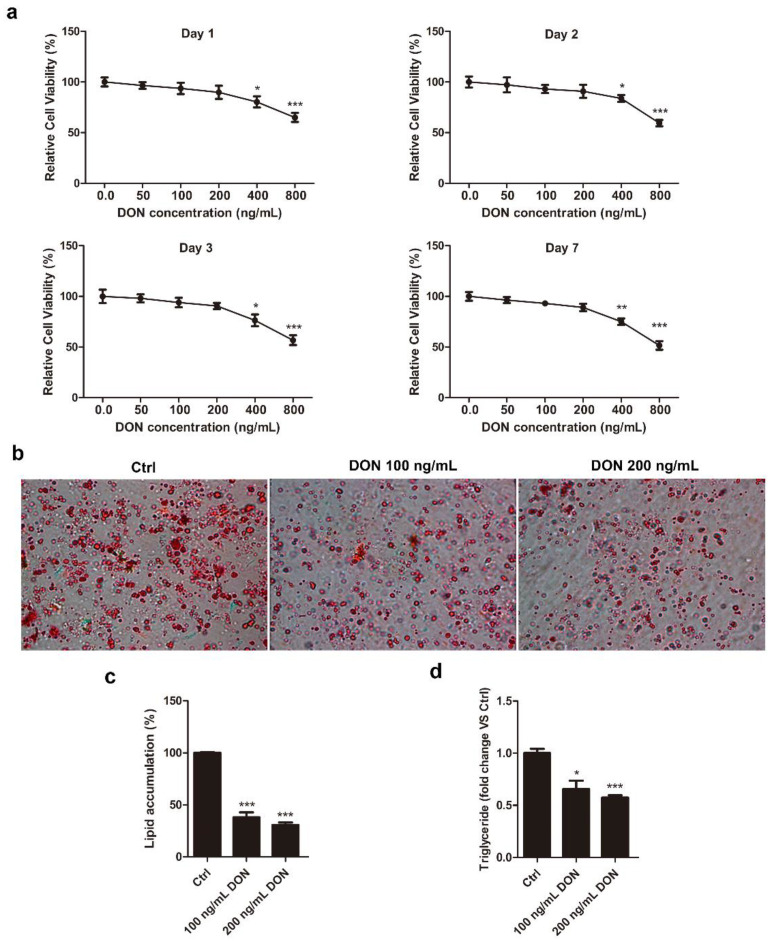
Effect of DON on adipogenesis in 3T3-L1 cells. (**a**) Cytotoxicity of DON in 3T3-L1 cells. The effect of DON on metabolic activity was examined by the CCK-8 assay. 3T3-L1 preadipocytes were treated with 0, 50, 100, 200, 400, or 800 ng/mL DON for 1, 2, 3, or 7 d, respectively. The values are expressed as percentages of control responses and were obtained from three independent experiments with six replications. (**b**) Lipid accumulation was visualized and quantified by Oil Red O staining. The cells were photographed at ×100 magnification, and morphological changes were assessed based on lipid accumulation with or without DON (100 and 200 ng/mL) on Day 7. (**c**) 3T3-L1 cells were treated with isopropanol, and lipid accumulation was measured according to the absorbance at 510 nm. Lipid accumulation in the control group cells was compared to that in the DON-induced cells. (**d**) DON inhibited TG accumulation in 3T3-L1 cells. 3T3-L1 preadipocytes were cultured in DMEM + FBS medium containing the differentiation cocktail, with or without DON (100 and 200 ng/mL) for 7 days. The results are representative of three independent experiments, and statistical significance is indicated by * *p* < 0.05, ** *p*  < 0.01, *** *p*  < 0.001.

3T3-L1 is a well-established cell line commonly used to study adipogenesis in vitro [33]. DON cytotoxicity was measured in 3T3-L1 cells by a CCK-8 assay. The cells were incubated with various concentrations of DON (50–800 ng/mL) for 1, 2, 3, or 7 d (Figure 1a). DON administration to 3T3-L1 cells at 50 and 100 ng/mL had little effect on cell proliferation, and that at 200 ng/mL caused less than 10% cell death at all treatment times. However, a more obvious decrease in cell viability was observed upon treatment with 400 and 800 ng/mL DON exposure, suggesting that its cytotoxicity was dose dependent. To minimize the toxicity of DON in the cells, we determined 100 ng/mL as the optimal maximal concentration in 3T3-L1 cells. 3T3-L1 preadipocytes were induced to differentiate into mature adipocytes and then treated with 100 and 200 ng/mL DON for 7 days. To investigate the effects of DON on intracellular lipid accumulation, the number of lipid droplets in adipocytes was determined by Oil Red O staining. The number of lipid droplets was clearly decreased in DON-induced cells compared to controls (Figure 1b,c). In addition, cells treated with 100 and 200 ng/mL DON displayed significant decreases in the basal levels of triglycerides (0.65- and 0.57-fold, respectively) (Figure 1d). DON had a dose-dependent, negative effect on both lipid droplets and TG levels. These data suggested that DON inhibited adipogenesis and had an apparent negative impact on 3T3-L1 adipocytes, even at relatively non-toxic concentrations. 

### 2.2. Low Expression Levels of Adipogenic Marker Genes in Differentiated 3T3-L1 Cells Treated with DON

**Figure 2:**
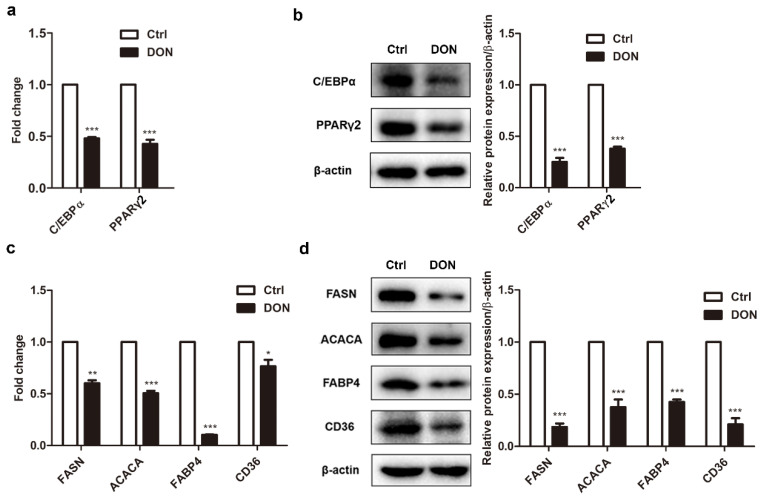
The effect of DON on the expression of adipogenic marker genes in 3T3-L1 cells. 3T3-L1 adipocytes were treated with 100 ng/mL DON during differentiation. The cells were harvested at the end of a 7-day differentiation. The expression of adipogenic marker genes was quantified by RT-PCR and Western blotting. (**a**,**c**) *C/ebpα*, *Pparγ2*, *Fasn*, *Acaca*, *Fabp4*, and *Cd36* mRNA levels in 3T3-L1 cells. (**b**,**d**) C/EBPα, PPARγ2, FASN, ACACA, FABP4, and CD36 protein levels in 3T3-L1 cells (left). Quantification of protein levels (right). The results are representative of three independent experiments, and statistical significance is indicated by * *p* <  0.05, ** *p* < 0.01, *** *p*  <  0.001.

Highly expressed during adipogenesis, C/EBPα and PPARγ2 coordinately regulate each other and play critical roles in the regulation of adipocyte phenotypes, as well as triglyceride accumulation [34]. To understand the molecular mechanisms underlying the DON-induced inhibition of adipogenesis, the expression of genes involved in control of adipogenesis were assessed in 3T3-L1 cells. Treatment with 100 ng/mL DON substantially decreased the mRNA and protein levels of C/EBPα and PPARγ2 vs. control (Figure 2a,b), implying that DON exposure affected adipogenesis by inhibiting the expression of two master regulators. The expression of other adipogenic marker genes was examined. As expected, the expression of two lipogenic enzymes, FASN and ACACA, and the fatty acid transport proteins, FABP4 and CD36, was significantly downregulated at both the mRNA and protein levels by DON administration. (Figure 2c,d). However, the expression of early adipogenic factors such as C/EBPβ and C/EBPδ, which regulate PPARγ2 expression, were not apparently affected by DON administration (Figure S1a,b). This suggested that DON exposure might affect adipogenesis by targeting the control of PPARγ2 expression.

### 2.3. Overexpression of PPARγ2 Attenuated DON-Induced Inhibition of Adipogenesis

**Figure 3:**
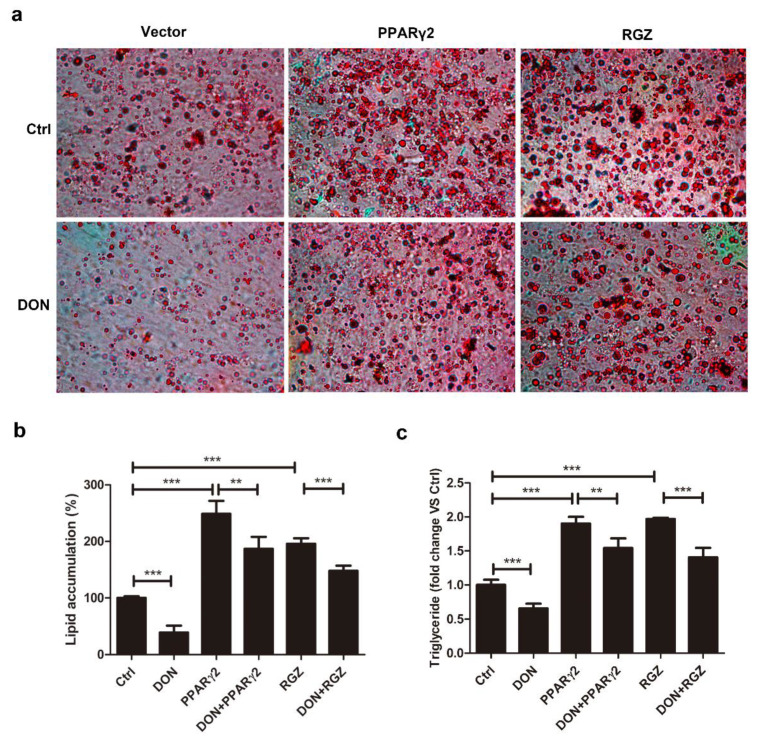
PPARγ2 overexpression attenuated the DON-induced inhibition of adipogenesis in 3T3-L1 cells. 3T3-L1 preadipocytes were transfected with PPARγ2 or treated with RGZ (1 μM) and then cultured in DMEM + FBS medium containing the differentiation cocktail with or without DON (100 ng/mL) for 7 days. (**a**) Oil Red O staining. The cells were photographed at ×100 magnification. (**b**) Quantification of lipid accumulation. (**c**) TG levels. The results are representative of three independent experiments, and statistical significance is indicated by ** *p* <  0.01, *** *p*  < 0.001.

PPARγ2 is the most important inducer of adipogenesis, and its expression in 3T3-L1 cells is strongly inhibited by DON. To verify whether the PPARγ2 pathway governed adipogenesis in DON-induced adipocytes, PPARγ2 was overexpressed in 3T3-L1 cells (Figure S2). As shown in Figure 3a,b, PPARγ2 overexpression significantly increased the number of lipid droplets and reduced the inhibitory effect of DON on the number of lipid droplets. A similar but less pronounced result was observed in cells treated with 1 μM rosiglitazone (RGZ), a PPARγ agonist (Figure 3a,b). PPARγ2 overexpression antagonized the DON-induced reduction of TG levels, slightly better than RGZ (Figure 3c). These results further indicated that DON downregulated PPARγ2 at the protein level, and even RGZ agonism was unable to fully restore its functions in DON-treated cells. Therefore, DON-induced downregulation of PPARγ2 prevented adipogenesis and the accumulation of lipid droplets during 3T3-L1 cell differentiation. 

### 2.4. Overexpression of PPARγ2 Attenuated DON-Induced Downregulation of Adipogenic Marker Genes

**Figure 4:**
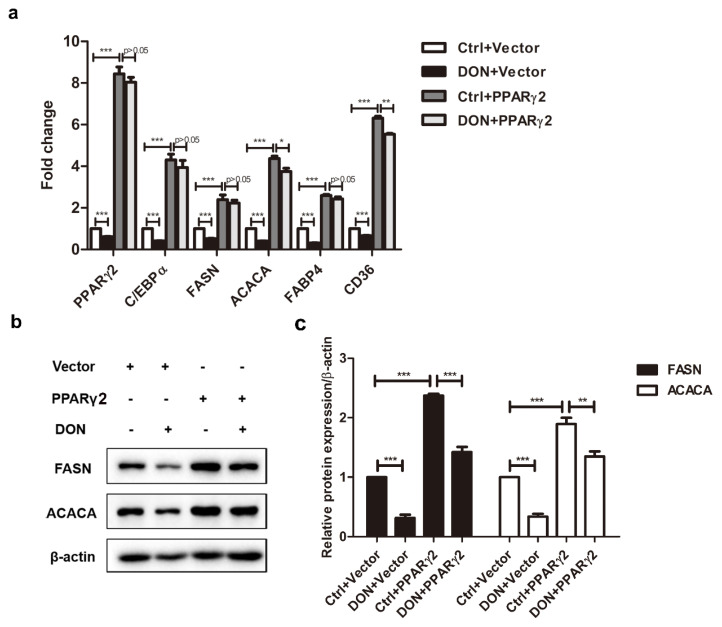
DON-induced downregulation of adipogenic marker genes was reversed by PPARγ2 overexpression in 3T3-L1 cells. Adipocyte cells were transfected with PPARγ2 or empty vector and treated with or without 100 ng/mL DON during differentiation. Total RNA or protein was isolated at the end of a 7-day differentiation. RT-PCR and Western blot analyses confirmed the expression of adipogenic marker genes. (**a**) *C/ebpα*, *Pparγ2*, *Fasn*, *Acaca*, *Fabp4*, and *Cd36* mRNA levels in 3T3-L1 cells. (**b**) FASN and ACACA protein levels in 3T3-L1 cells. (**c**) Quantification of protein levels. The DON, PPARγ2 overexpression, and DON + PPARγ2 overexpression groups normalized by the control group. The results are representative of three independent experiments, and statistical significance is indicated by * *p* <  0.05, ** *p* <  0.01, *** *p* <  0.001.

We investigated whether the expression of other adipogenic marker genes controlled by PPARγ2 and downregulated by DON treatment was restored in PPARγ2-overexpressing cells. PPARγ2 overexpression significantly upregulated the mRNA levels of the adipogenic marker genes *C/ebpα*, *Fasn*, *Acaca*, *Fabp4*, and *Cd36*. The inhibitory effect of DON on these genes was apparently abrogated (Figure 4a). PPARγ2 overexpression also restored the expression of FASN and ACACA at the protein level in DON-induced 3T3-L1 adipocytes (Figure 4b,c). These results suggested that the DON-induced inhibition of adipogenic marker genes was counteracted by PPARγ2 overexpression, showing that the PPARγ2 pathway played a crucial role in the DON-induced inhibition of adipogenesis.

### 2.5. DON Decreases WAT Size and TG Levels in BALB/c Mice

**Figure 5:**
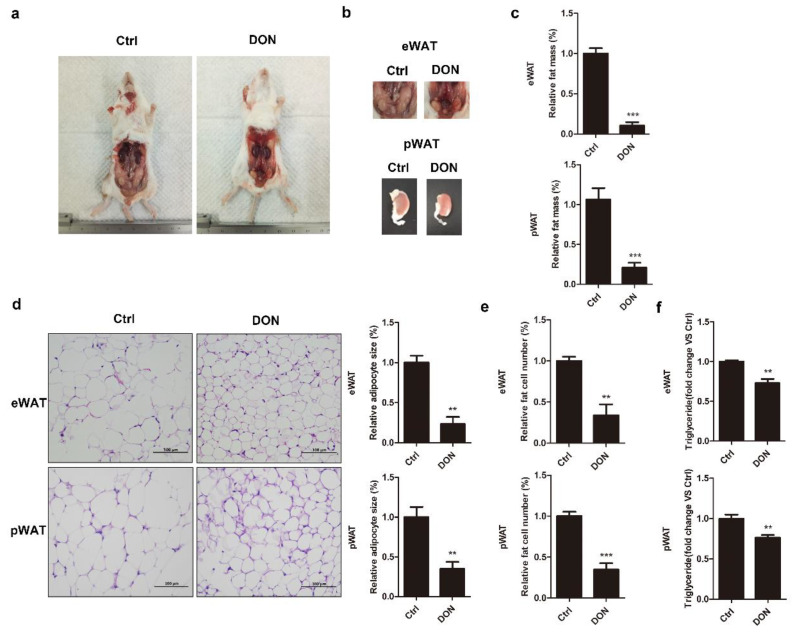
The effect of DON on adipogenesis in mice. Five-week-old SPF male BALB/c mice were randomized into two groups (*n* = 5/group)—control group and DON group (3 mg/kg/day)—for four weeks. (**a**) Representative pictures of mice. (**b**) The volume of epididymal eWAT and pWAT. (**c**) The weights of eWAT and pWAT in DON-induced mice compared to those in the control mice. (**d**) Histological analysis of eWAT and pWAT after staining with hematoxylin and eosin (H&E), followed by microscopic analysis. Scale bar is 100 µm (left). Average cell size in DON-induced mice compared to that in the control group (right). (**e**) The relative cell numbers of eWAT and pWAT in DON-induced mice compared to those in the control mice. (**f**) The TG level in DON-induced mice compared to that in the control group. Data are presented as means ± SD, *n* = 5. The results are representative of three independent experiments, and statistical significance is indicated by ** *p* < 0.01, *** *p*  < 0.001.

To further confirm the effect of DON on adipogenesis in vivo, the body weights of mice were measured weekly for 28 days. Decreased body weight gain was observed after Day 21 in DON-induced mice and was most obvious on Day 28, compared to the control group (Figure S3a). The mice were sacrificed on Day 28, and then, their epididymal white adipose tissue (eWAT) and perirenal white adipose tissue (pWAT) were examined, showing a drastically reduced volume after DON administration (Figure 5a,b). DON exposure markedly reduced epididymal and perirenal fat mass (10.11% and 20.43%) compared to those of the control group (Figure 5c). Histological analysis of the adipocyte tissue showed that, in DON-induced mice, the size of cells in the epididymal and perirenal adipose tissues was significantly decreased compared to that of those of the control mice (Figure 5d). DON exposure also reduced the epididymal and perirenal fat cell numbers compared to those in the control group (Figure 5e). The triglyceride content, reflecting the extent of lipid storage, was also decreased in the DON-induced animals (Figure 5f). We next determined that the mRNA levels of *caspase 3* and *caspase 9* were upregulated by DON treatment in the mice’s livers and kidneys, suggesting that DON not only inhibited adipogenesis in mouse adipose but also caused the apoptosis of mouse livers and kidneys (Figure S3b).

### 2.6. Low Expression Levels of Adipogenic Marker Genes in Mice Treated with DON

**Figure 6:**
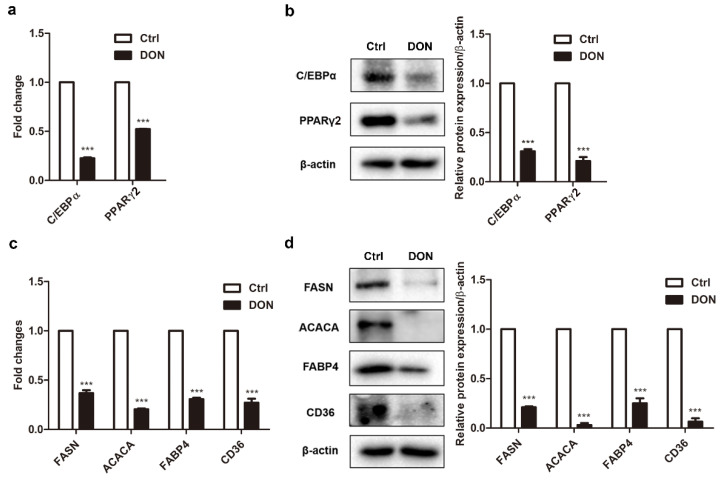
The effect of DON on the expression of adipogenic marker genes in mice (*n* = 5). (**a**,**c**) *C/ebpα*, *Pparγ2*, *Fasn*, *Acaca*, *Fabp4*, and *Cd36* mRNA levels in mice. (**b**,**d**) C/EBPα, PPARγ2, FASN, ACACA, FABP4, and CD36 protein levels in mice (left). Quantification of protein levels (right). The results are representative of three independent experiments, and statistical significance is indicated by *** *p*  <  0.001.

The DON exposure inhibited adipogenesis in the mice, consistent with the result in 3T3-L1 cells. It could be argued that the decreased body weight gain in the mice consequently inhibited adipogenesis in the early stage because of insufficient nutrient uptake. However, the unchanged expression levels of C/EBPβ and C/EBPδ (Figure S1c,d) suggested that early adipogenesis was not affected. Instead, a significant decrease in the PPARγ2 pathway indicated that DON exposure only inhibited the intermediate and late stages. In the mice exposed to DON, the mRNA and protein levels of the adipogenic marker genes *C/ebpα*, *Pparγ2*, *Fasn*, *Acaca*, *Fabp4*, and *Cd36* were significantly decreased compared to those in the control group (Figure 6a–d). These results indicated that DON did not affect the early stage of adipogenesis but effectively inhibited the intermediate and late stages through the downregulation of adipogenic marker genes, both in vivo and in vitro.

### 2.7. DON Suppressed the Expression of PPARγ2 by Inhibiting the Enrichment of H3K4me2 and H3K4me3 in 3T3-L1 Cells

As mentioned above, DON inhibited adipogenesis via the inhibition of PPARγ2 expression. However, the mechanism behind this in 3T3-L1 cells remains to be elucidated. It is well recognized that DON activates mitogen activated protein kinases (MAPK) subfamily members [35,36,37]. We speculated that DON stimulated the phosphorylation of PPARγ2 by activating the MAPK pathways. Indeed, DON significantly upregulated p38 and JNK but not ERK (Figure S4a). However, the DON-induced activation of MAPK did not affect significantly the phosphorylation of PPARγ2 (Figure S4b), suggesting that DON regulated PPARγ2 in a MAPK-independent manner. To determine if the inhibitory effect of DON on *Pparγ2* was related to transcription, we employed the dual luciferase assay to analyze the promoter activity of *Pparγ2* in HEK293T cells. A 1.0 kb segment of *Pparγ2* promoter DNA was cloned upstream of the firefly luciferase gene. The quantitative analysis referred to the promoter strength of PPARγ2 normalized by the luciferase activities relative to that obtained with the empty vector. However, the relative luciferase activity showed no significant change upon DON treatment in comparison with the control, which was treated with sterile water (DON solvent) (Figure S5). These results suggested that DON had no effect on the promoter activation of *Pparγ2* at the transcriptional level. 

**Figure 7:**
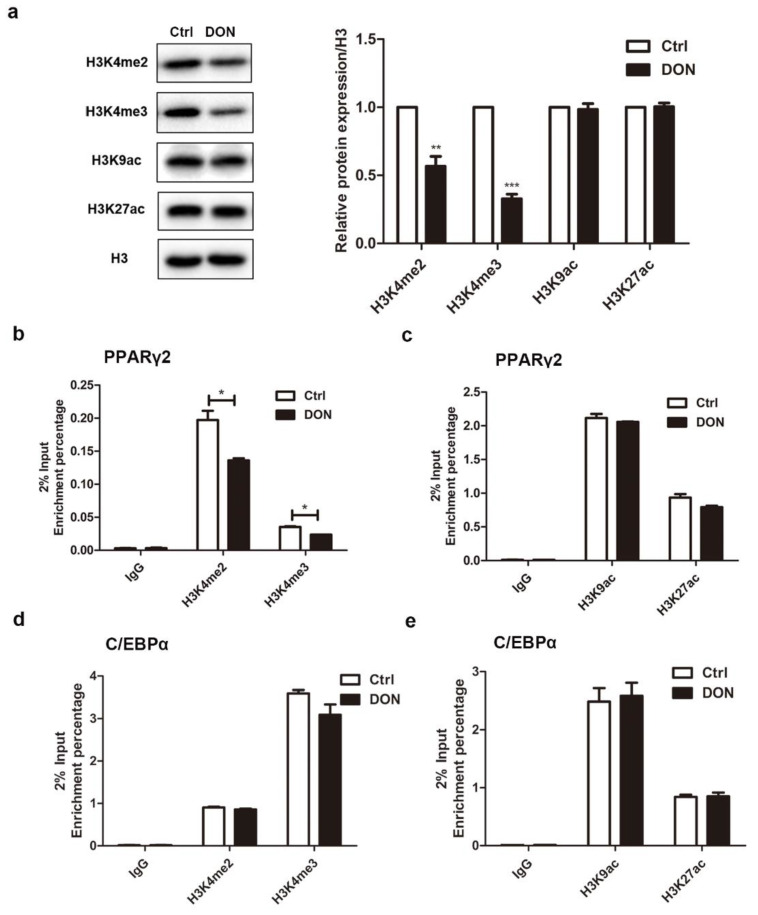
DON regulated the expression of PPARγ2 through H3K4me2 and H3K4me3. (**a**) 3T3-L1 cells were differentiated and treated with or without DON (100 ng/mL) for 7 days. The expression of H3K4me2, H3K4me3, H3K9ac, and H3K27ac was quantified by Western blotting in 3T3-L1 cells (left). Quantification of protein levels (right). (**b**) The 3T3-L1 cells were differentiated and treated with or without DON (100 ng/mL) for 7 days. The cells were prepared for ChIP assays using the indicated antibodies. The binding occupancy was monitored by qPCR using primer sets for the targeted promoter. ChIP assays of H3K4me2 and H3K4me3 on *Pparγ2* promoter. (**c**) ChIP assays of H3K9ac and H3K27ac on *Pparγ2* promoters. (**d**) ChIP assays of H3K4me2 and H3K4me3 on *C/ebpα* promoters. (**e**) ChIP assays of H3K9ac and H3K27ac on *C/ebpα* promoters. The data are expressed as the fold enrichment relative to that of IgG normalized to that of a nonbinding region. The results are representative of three independent experiments, and statistical significance is indicated by * *p* < 0.05, ** *p*  < 0.01, *** *p*  < 0.001.

Recent studies suggest that histone modifications play critical roles in regulating adipogenic gene expression and adipogenesis [38]. We investigated whether epigenetic changes mediated the DON-induced downregulation of PPARγ2. The demethylation and trimethylation levels of Histone 3 at lysine 4 were apparently decreased upon DON treatment, but there were no apparent changes in the acetylation of H3K9 and H3K27 (Figure 7a). ChIP-qPCR analysis further confirmed that the enrichment of H3K4me2 and H3K4me3 at the promoter of *Pparγ2* decreased upon DON treatment (Figure 7b), while that of H3K9ac and H3K27ac was not significantly changed (Figure 7c). The enrichment of H3K4me2, H3K4me3, H3K9ac, and H3K27ac at the promoter of *C/ebpα* did not significantly change upon DON treatment (Figure 7d,e). These results suggested that DON suppressed the expression of PPARγ2 by reducing the enrichment of H3K4me2 and H3K4me3 at the promoter of *Pparγ2*. Therefore, the decreased levels of H3K4me2 and H3K4me3 at the *Pparγ2* promoter locus may account for the repression of adipogenesis upon DON treatment.

## 3. Discussion

DON-contaminated food (particularly wheat, maize, barley, and their byproducts) is harmful for animal and human health. DON causes ribosomal stress toxicity, oxidative stress, and immune disorders [7,39,40]. We confirmed that DON dramatically decreased lipid accumulation and TG levels in 3T3-L1 cells. Similar effects were also observed in vivo, where DON decreased the volume and sizes of eWAT and pWAT, and reduced TG content. Our current study suggested that the master regulator PPARγ2 was probably a novel DON target in the toxicosis of DON, similar to β-catenin that we previously identified [41]. More importantly, the interruption of the intermediate and late stages of adipogenesis by the low dose of DON might be one of the mechanisms behind the decreased weight gain in the animals fed DON-contaminated feedstuffs, in addition to feed refusal, loss of appetite, acute vomiting, and diarrhea.

Adipogenesis is a process for adipocyte differentiation and lipid accumulation [42,43]. Preadipocyte differentiation into mature adipocytes is a tightly controlled and stepwise process that involves the expression changes of serial adipogenic genes and expansion of intracellular lipid droplets. The sequential activations of adipocyte-related transcription factors govern the differentiation of preadipocytes into mature adipocytes [44]. Our results show that DON exposure suppressed the expression of PPARγ2 and further inhibited the expression of other adipogenic marker genes—*Fasn*, *Aacac*, *Fabp4*, and *Cd36*—which were required for adipogenesis in both cells and mice. In 3T3-L1 cells, DON globally decreased the di-/trimethylation level of H3K4 and, especially at the *Pparγ2* promoter locus, suppressed the transcription of *Pparγ2*, consequently inhibiting adipogenic marker expression during the intermediate and late stages of adipogenesis. 

PPARγ2 is considered one of the most important regulators of adipogenesis [45], and a complicated mutual modulation occurs between PPARγ2 and C/EBPα. They promote each other’s expression and cooperate to activate the expressions of thousands of genes involved in late adipogenesis [14]. A recent study reported that another type B trichothecene, fusarenon-X, is a stronger inducer of intestinal inflammation, and PPARγ is also downregulated after its administration [46]. In HCT-8 cells, DON may suppress PPARγ expression via CCAAT/enhancer-binding protein homologous protein (CHOP). The transcription level of *CHOP* is enhanced by DON, and the high expression of CHOP further downregulates the expression of PPARγ [47]. All of these studies imply that type B trichothecenes negatively regulate PPARγ. In our study, we also confirmed that DON downregulated the expression of *Pparγ2* both in vitro and in vivo. DON also markedly decreased the expression of two rate-limiting enzymes in lipogenesis, FASN and ACACA, which are involved in de novo fatty acid synthesis [48]. The expression of FABP4 and CD36, two proteins involved in cytoplasmic fatty acid transport for metabolic processing or storage, was also decreased. Conclusively, DON inhibited the expression of PPARγ2 and, subsequently, C/EBPα, reducing the expression of other adipogenic marker genes in adipocytes. Because the expression of FASN, ACACA, FABP4, and CD36 is known to be controlled by PPARγ [32,49,50,51], we reasoned that DON suppressed adipocyte differentiation by acting on the PPARγ2 pathway. PPARγ2 overexpression reversed the suppression by DON and induced lipid accumulation, consistent with a previous report [19]. RGZ treatment rescued the lipid accumulation in DON-treated 3T3-L1 cells but less so than PPARγ2 overexpression. This further confirmed that DON exposure decreased the protein level of PPARγ2. During the early stage of adipogenesis, C/EBPβ and C/EBPδ activate the expression of C/EBPα, PPARγ, and probably other adipogenic genes [52]. Interestingly, DON did not affect the expression of C/EBPβ and C/EBPδ, in vitro or in vivo. This suggests that DON possibly has no regulatory effect on the early stage of adipogenesis but regulates PPARγ2 expression in the intermediate and late stages of adipogenesis. More importantly, this indicates that lower nutrient uptake due to the feed refusal, vomiting, or diarrhea caused by DON exposure does not account for DON-induced adipogenesis suppression, otherwise the expression of C/EBPβ or C/EBPδ would also be changed [53,54]. Therefore, the interruption of adipogenesis independently of malnutrition resulting from feed refusal, vomiting, or diarrhea might explain the decrease of weight gain by DON exposure in animals.

The molecular basis of the DON-induced effects on PPARγ2 remains unclear. The MAPK-mediated phosphorylation of PPARγ2 has been proven to inhibit PPARγ2 activity [55]. ERK phosphorylates PPARγ2, which results in the inactivation of PPARγ2 activity [56]. Several studies have already shown that DON activates MAPK subfamily members [35,36,37]. However, we found that DON regulated the expression of PPARγ2 in a MAPK-independent manner, because DON exposure did not change PPARγ2 phosphorylation. Consistently, our results showed that DON enhanced the phosphorylation of p38 and JNK but not ERK, which had been reported to function in PPARγ2 phosphorylation. The transcriptional control of PPARγ expression has been extensively studied. Nevertheless, the promoter activity of *Pparγ2* shows no significant change upon DON treatment. Histone modifications play important roles in regulating the expression of adipogenic marker genes and adipogenesis [38]. Those resulting in transcriptional activation or repression are observed near the *Pparγ2* promoter in adipocytes [24]. It has been reported that the enrichment of H3K9ac and H3K27ac in promoter regions is affected by DON administration [57]. Interestingly, we found that the inhibitory effect of DON on the expression of PPARγ2 was mediated by inhibiting H3K4me2 and H3K4me3 enrichment instead of that of H3K9ac and H3K27ac at the promoter of *Pparγ2* in 3T3-L1 cells. More importantly, H3K4me2 and H3K4me3 were globally repressed in 3T3-L1 cells by DON administration, possibly based on cell tropism. Such changes in adipocytes warrant further study. H3K4me2 is typically found at the enhancer element, while H3K4me3 is observed at promoter regions. Di-/trimethylation is associated with the histone lysine (K)-specific methyltransferase 2C/2D (MLL3/MLL4) [58]. The histone H3K4 methyltransferase MLL4 enhances the expression of PPARγ2 [59]. The idea that MLL3/4 may be downregulated in the inhibitory effect of DON on PPARγ2 expression remains to be further addressed. In addition, PPARγ2′s functions are also regulated by various post-translational modifications (PTMs) including SUMOylation, ubiquitination, acetylation, and O-GlcNAcylation, at numerous sites [60]. Further research is required to elucidate the mechanisms affecting the expression or activity of PPARγ2 in DON treatment. 

Our study demonstrates that DON—a type B trichothecene—inhibits adipogenesis, a novel toxic effect, even at low doses. In particular, we show that the pivotal regulator PPARγ2 is targeted by DON, which inhibits its expression during the intermediate and late stages of adipogenesis. We proposed that the di-/trimethylation level of H3K4 mediates the DON-induced downregulation of PPARγ2. The inhibition of adipogenesis by DON may have consequences for animal health and highlights the potential risk of the long-term consumption of food contaminated by DON in animals and humans.

## 4. Materials and Methods

### 4.1. Cell Culture 

3T3-L1 (ATCC CL-173) cells were purchased from American Type Culture Collection (ATCC, Manassas, VA, USA) and cultured in Dulbecco’s modified Eagle’s medium (Thermo Fisher, Waltham, MA, USA) supplemented with 10% fetal bovine serum (Biological Industries, Kibbutz Beit Haemek, Israel) in a 37 °C incubator containing a 5% CO_2_ atmosphere. For adipocyte differentiation, 3T3-L1 preadipocytes were trypsinized and plated in a 12-well plate. Cells were cultured for an additional day after reaching confluency, and 10 mg/mL insulin (Sangon Biotech, Shanghai, China), 1 µM dexamethasone (Sigma, St. Louis, MO, USA), and 0.5 mM 3-isobutyl-1-methylxanthine (Sangon Biotech, Shanghai, China) were added to the culture medium for 2 days. The medium was then replaced with the basic culture medium containing only 10 mg/mL insulin for another 2 days. From Days 4 to 7, the cells were maintained in basic culture medium and were additionally administrated 100 ng/mL DON and 1 µM rosiglitazone (RGZ) (MedChemExpress, Princeton, NJ, USA) from Days 0 to 7 during differentiation. The DON and RGZ were replenished every two days.

### 4.2. Construction of Plasmids, Adenovirus Transfection, and Luciferase Activity Detection

Based on NCBI GenBank, we amplified the open reading frame (ORF) of mouse PPARγ2 (GenBank Accession Number: NC_000072.6) without a stop codon by PCR. cDNA from the reverse transcription of RNA from 3T3-L1 adipocytes was used as a template. The pSIN-cFlag-Pur vector was digested with the restriction enzyme SnaB I (NEB, Beijing, China). The expression vector pSIN-PPARγ2-cFlag-Pur-containing mouse PPARγ2 was constructed by subcloning the corresponding cDNA of PPARγ2 into N-terminal Flag-tagged pSIN-cFlag-Pur (Invitrogen, Rockville, MD, USA) using a ClonExpress II One-Step Cloning Kit (Vazyme, Nanjing, China) according to the manufacturer’s instructions. Adenoviruses encoding PPARγ2 were generated by the transfection of the plasmids containing the expression vector of a PPARγ2 construct, psPAX2 (Invitrogen, Rockville, MD, USA), pMD2.G (Invitrogen, Rockville, MD, USA), and an empty vector (pSIN-cFlag-Pur) as the control. Finally, puromycin was used to select for the stably expressing cells. 

According to NCBI GenBank, the 5′-flanking region from −1000 to −1 of the construct (the translational start site was designated +1) of mouse *Pparγ2* was amplified by using mouse genomic DNA from 3T3-L1 cells by PCR. The Sac I (TaKara, Qingdao, China)- and Xho I (Takara, Qingdao, China)-digested PCR fragments were inserted upstream of pGL3-Basic (Promega Corp., Madison, WI, USA) to generate the luciferase reporter constructs. HEK293T cells were seeded into 24-well plates and cultured in DMEM until reaching 80% confluence. For the transfection reporter constructs of PPARγ2, 0.6 μg of firefly luciferase reporter plasmids and 0.06 μg of *Renilla* luciferase reporter plasmid (pRL-TK) (Promega Corp., Madison, WI, USA) were incubated with 1.5 μL of Lipofectamine 3000 (Invitrogen, CA, USA). The transfected cells were incubated for 24 h, and then, the transfected cells were treated with DON or sterile water for another 24 h. The cells were lysed, and the luciferase reporter activity was measured with the dual luciferase reporter assay system (Promega Corp., Madison, WI, USA) according to the manufacturer’s instructions. The strength of the promoter’s transcription was represented by the relative luciferase activity, and the firefly luciferase activity in each construct was normalized to the *Renilla* luciferase activity as a transfection reference. All experiments were performed three times independently. The specific primer sets used for the construction of the expression vector are listed in Table S1.

### 4.3. Cell Viability Assay

3T3-L1 preadipocytes were grown in a 96-well plate at an initial density of 1.5×10^4^ cells/cm^2^ in the presence of DON at different concentrations (50, 100, 200, 400, or 800 ng/mL) for 1, 2, 3, or 7 d. Cell viability was determined using a Cell Counting Kit (CCK-8) (Ye Sen, Guangdong, China). The absorbance at 450 nm was measured with a spectrophotometer using SoftMax Pro 7.0 (Molecular Devices, Sunnyvale, CA, USA).

### 4.4. Oil Red O Staining 

The mature adipocytes were grown on a coverslip and fixed with 10% formalin (Guangzhou Dingguo Biology, Guangdong, China) at room temperature for 1 h. After fixation, the cells were washed once with 60% isopropyl alcohol (Sangon Biotech, Shanghai, China). Oil Red O solution (Sangon Biotech, Shanghai, China) was prepared according to the manufacturer’s instructions and added to the plate for 1 h. The coverslips were then visualized under a microscope. The cell culture plates were treated with isopropanol (Sangon Biotech, Shanghai, China), and lipid accumulation was determined according to absorbance at 510 nm.

### 4.5. TG Colorimetric Assay

The mature adipocytes were washed with ice-cold PBS, collected in 200 µL of cold sonication buffer (25 mM Tris buffer containing 1 mM EDTA, pH 7.4) using a cell scraper, and then sonicated. The supernatants were used to measure intracellular TG levels using a TG kit (Jiancheng Institute of Biotechnology, Nangjing, China), according to the manufacturer’s instructions. Epididymal white adipose tissues (eWAT) and perirenal white adipose tissues (pWAT) were homogenized in ethanol solution (9:1 V/m) before centrifugation, and the supernatants were used to measure TG levels using a TG kit.

### 4.6. Real-Time PCR (qPCR)

Total RNA was isolated from eWAT isolated adipocytes, liver, kidney, or 3T3-L1 adipocytes using TRIzol reagent (Thermo Fisher, Waltham, MA, USA) under RNase-free conditions. Reverse transcription and qPCR were performed in a simultaneous reaction using Hifair TM III One Step RT-qPCR Probe Kit (Ye Sen, Guangdong, China) according to the manufacturer’s instructions. All RT-qPCR reactions were carried out in a 96-well plate format on a CFX Connect real time qPCR machine (Bio-Rad Laboratories, Hercules, CA, USA). The amplification protocol included an initial denaturation step of 10 min at 95 °C followed by 40 cycles consisting of denaturation for 15 s at 95 °C, annealing for 1 min at 55 °C, and extension for 1 min at 72 °C, followed by melting curve analysis. Relative expression was normalized to β-actin using the 2^−ΔΔCt^ method. The amplification of specific transcripts was confirmed by obtaining melting curves between 55 and 95 °C. The specific primer sets used for qPCR are listed in Table S2.

### 4.7. Western Blotting

Cells were lysed in NETN (20 mM Tris-HCl pH 8.0, 100 mM NaCl, 1 mM EDTA, 0.5% NP-40) supplemented with a protease inhibitor cocktail (Bimake, Houston, TX, USA). Protein concentrations were determined using a BCA Protein Assay Kit (Guangzhou Dingguo Biology, Guangdong, China). All protein samples were separated by sodium dodecylsulphate–polyacrylamide gel electrophoresis (Bio-Rad Laboratories, Hercules, CA, USA) and transferred to PVDF membranes (Merck Millipore, Burlington, MA, USA), which were blocked with 5% skim milk (Guangzhou Dingguo Biology, Guangdong, China) and hybridized with primary antibodies. Horseradish peroxidase-conjugated donkey anti-goat IgG, goat anti-rabbit IgG, and rabbit anti-mouse IgG (Cell Signaling Technology, Danvers, MA, USA) were used as secondary antibodies. Signals were detected by using the ChemiDoc MP system (Bio-Rad Laboratories, Hercules, CA, USA) with the BeyoECL Star (Beyotime, Beijing, China) or the Efficient Chemiluminescence Kit (Genview, Guangdong, China). The bands were quantified using Image Lab 4.1 (Bio-Rad Laboratories, Hercules, CA, USA).

Anti-PPARγ (sc-7273, Santa Cruz Biotechnology, Dallas, TX, USA), anti-C/EBPα (sc-9315X, Santa Cruz Biotechnology, Dallas, TX, USA), anti-C/EBPβ (D155298, Sangon Biotech, Shanghai, China), anti-C/EBPδ (D160164, Sangon Biotech, Shanghai, China), anti-ACACA (D155300, Sangon Biotech, Shanghai, China), anti-FASN (D262701, Sangon Biotech, Shanghai, China), anti-FABP4 (D120618, Sangon Biotech, Shanghai, China), anti-CD36 (D161529, Sangon Biotech, Shanghai, China), anti-p-PPARγ (bs-3737R Bioss, Beijing, China), anti-H3K4me2 (9725S, Cell Signaling Technology, Danvers, MA, USA), anti-H3K4me3 (9751S, Cell Signaling Technology, Danvers, MA, USA), anti-H3K9ac (9649S, Cell Signaling Technology, Danvers, MA, USA), anti-H3K27ac (8173S, Cell Signaling Technology, Danvers, MA, USA), anti-H3 (4620S, Cell Signaling Technology, Danvers, MA, USA), anti-ERK (AF1051, Beyotime Biotechnology, Shanghai, China), anti-p-ERK (AF1891, Beyotime Biotechnology, Shanghai, China), anti-JNK (AF1048, Beyotime Biotechnology, Shanghai, China), anti-p-JNK (AF1762, Beyotime Biotechnology, Shanghai, China), anti-p38 (AF7668, Beyotime Biotechnology, Shanghai, China), anti-p-p38 (AM063, Beyotime Biotechnology, Shanghai, China), and anti-β-actin (R4967S, Cell Signaling Technology, Danvers, MA, USA) antibodies were used for Western blot analysis.

### 4.8. Chromatin Immunoprecipitation (ChIP) Assays

Chromatin immunoprecipitation assays were performed using the ChIP Assay Kit (Millipore, Billerica, Massachusetts, USA). In brief, 3T3-L1 cells were fixed with 1% formaldehyde for 10 min at 37 °C to form DNA–protein crosslinks and quenched with 0.125 M glycine. Chromatin was isolated by the addition of lysis buffer. Each sample was sonicated on ice and then incubated with antibody at 4 °C overnight. The PCR quantitation of precipitated genomic DNA relative to input was performed in triplicate using a qPCR Probe Kit (Ye Sen, Guangdong, China) according to the manufacturer’s instructions. The sequences of the primers are listed in Table S3. The antibodies used were as follows: anti-IgG (R3900S, Cell Signaling Technology, Danvers, MA, USA), anti-H3K4me2, anti-H3K4me3, anti-H3K9ac, and anti-H3K27ac.

### 4.9. Experimental Animals

All experimental procedures were performed in conformance with the guidelines on animal experiments provided by the Institutional Care Committee of South China Agricultural University (Permit No.01952236, Date: 25 June 2019) (Guangzhou, Guangdong, China). Five-week-old specific-pathogen-free (SPF) male BALB/c mice were purchased from the Guangdong Medical Laboratory Animal Center (GDMLAC, Foshan, China). All the animals were fed with a standard diet (AIN-93M). The mice were housed in the animal facility under standard conditions (12/12 h light–dark cycle, 50 ± 15% humidity, 22 ± 2 °C temperature) for one week and then randomly assigned to two groups based on intragastric administration (*n* = 5/group): control group and DON (Sigma, St. Louis, MO, USA) group. The mice in the control group were administered 100 µL of sterile water. The mice in the DON group were administered DON at a dosage of 3.0 mg/kg body weight (100 µL) as in a previous study [61]. All the administrations were performed daily by gavage for four weeks. Body weight was recorded once a week. At the end of the administration period, the mice were sacrificed by cervical dislocation. The WAT, liver, and kidney were separated and weighed from each mouse, immediately frozen in liquid nitrogen, and stored at −80 °C until use.

### 4.10. Histological Analysis

For histological analysis, the adipocyte tissue was subsequently fixed in 4% *w*/*v* paraformaldehyde/PBS and embedded in paraffin, as previously described [62]. Hematoxylin and eosin (HE) staining was performed and visualized using a microscope. The size of the adipose tissue was measured using the Image J software with Adipocytes Tools plugins.

### 4.11. Statistical Analysis

For each experiment, three replicates were performed. Statistical differences between experimental groups were evaluated by one-way analysis of variance (ANOVA) with the least significant difference (LSD) test. All statistical analyses were performed using SPSS 20.0 (IBM, New York, USA). Statistical significance was defined by * *p* < 0.05, ** *p* < 0.01, *** *p* < 0.001.

## Supplementary Materials

Supplementary materials can be found at https://www.mdpi.com/1422-0067/21/17/6300/s1. Figure S1: The effect of DON on the expressions of C/EBPβ and C/EBPδ, both in vitro and in vivo. Figure S2: PPARγ2 overexpression in 3T3-L1 cells. Expression vector containing mouse PPARγ2 was constructed by subcloning the corresponding cDNA into N-terminal Flag-tagged pSIN-cFlag-Pur. Figure S3: The effect of DON on the change in body weight, liver function, and renal function. Figure S4. The effect of DON on the expression of MAPK and p-PPARγ2 in 3T3-L1 cells. Figure S5: Analysis of the *Pparγ2* promoter in HEK293T cells treated with DON. Table S1: Primers for construction of expression vector. Table S2: Primers for quantitative real-time PCR. Table S3: Primers for ChIP analysis.

## Abbreviations

ACACA	Fatty acid synthesis acetyl-CoA carboxylase alpha
bZIP	Basic region leucine zipper
CD36/FAT	Fatty acid translocase
C/EBPα	CCAAT/enhancer-binding proteins alpha
CHOP	CCAAT/enhancer-binding protein homologous protein
DON	Deoxynivalenol
eWAT	Epididymal white adipose tissue
FABP4	Fatty acid binding protein 4
FASN	Fatty acid synthase
HE	Hematoxylin and eosin
MAPK	Mitogen activated protein kinases
MLL3/MLL4	Lysine (K)-specific methyltransferase 2C/2D
PPARγ	Peroxisome proliferator-activated receptors gamma
PTMs	Post-translational modifications
pWAT	Perirenal white adipose tissue
RGZ	Rosiglitazone
SPF	Specific-pathogen-free
TG	Triglycerides
