# Supplementary material for: Deoxynivalenol Exposure Suppresses Adipogenesis by Inhibiting the Expression of Peroxisome Proliferator-Activated Receptor Gamma 2 (PPARγ2) in 3T3-L1 Cells
Source: Int J Mol Sci. 2020 Aug 31;21(17):6300. doi: 10.3390/ijms21176300 (PMC7504378; doi:10.3390/ijms21176300)
Supplement: Supplementary file 1 [file ijms-21-06300-s001.pdf]

Supplementary Materials

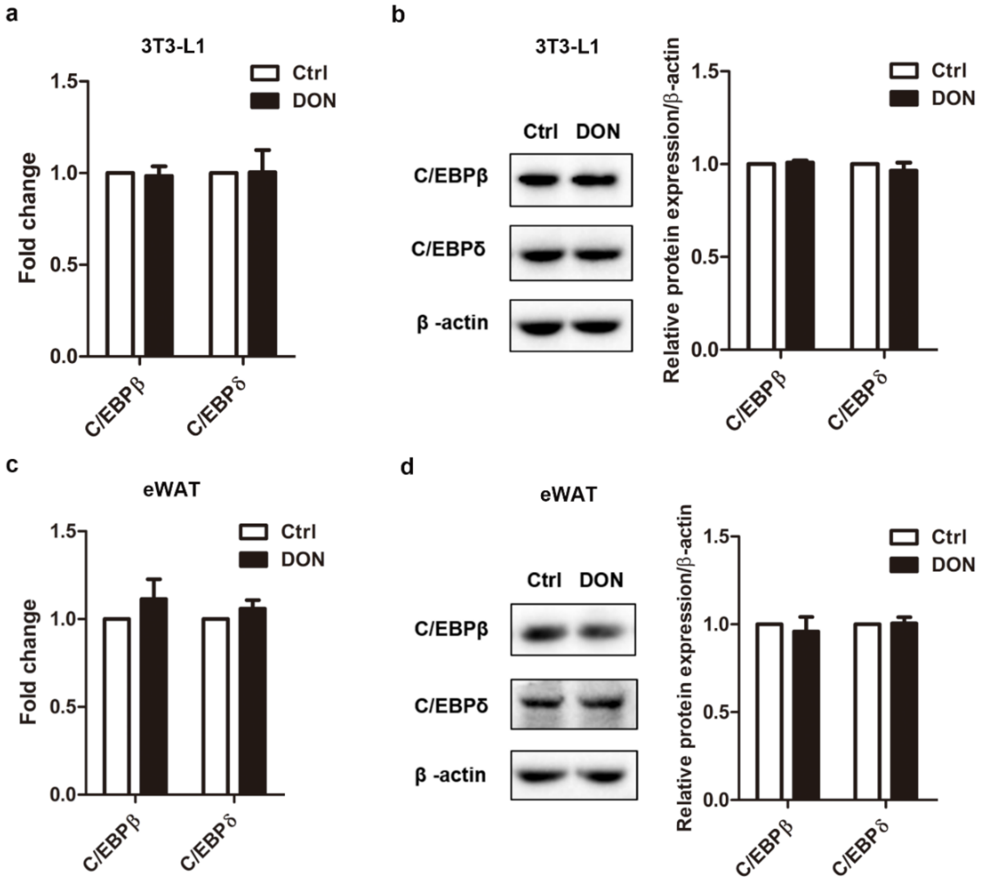

Figure S1. The effect of DON on the expressions of C/EBPβ and C/EBPδ, both in vitro and in vivo. (**a**) 3T3-L1 cells were differentiated and treated with or without DON (100 ng/mL) for 1 day. The mRNA levels of C/ebpβ and C/ebpδ were determined by RT-PCR. (**b**) The protein levels of C/EBPβ and C/EBPδ were determined by Western blotting (left). Quantification of protein levels (right). (**c**) Five-week-old SPF male BALB/c mice were randomized into two groups (n = 5/group)—the control group and DON group (3 mg/kg/day)—for four weeks. The mRNA levels of C/ebpβ and C/ebpδ were determined by RT-PCR in mice. (**d**) The protein levels of C/EBPβ and C/EBPδ were determined by Western blot in mice (left). Quantification of protein levels in mice (right). The results are representative of three independent experiments.

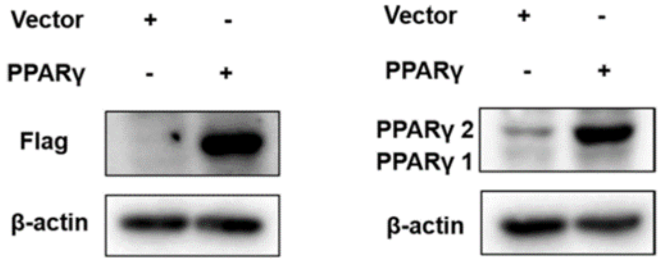

Figure S2. PPARγ2 overexpression in 3T3-L1 cells. Expression vector containing mouse PPARγ2 was constructed by subcloning the corresponding cDNA into N-terminal Flag-tagged pSIN-cFlag-Pur. Adipocyte cells were transfected with pSIN-cFlag-PPARγ2-Pur or empty vector. Total protein was isolated at the end of a 7 day differentiation. Western blot analyses quantified the PPARγ protein levels with antibodies against Flag (left) and PPARγ (right), respectively. The results are representative of three independent experiments.

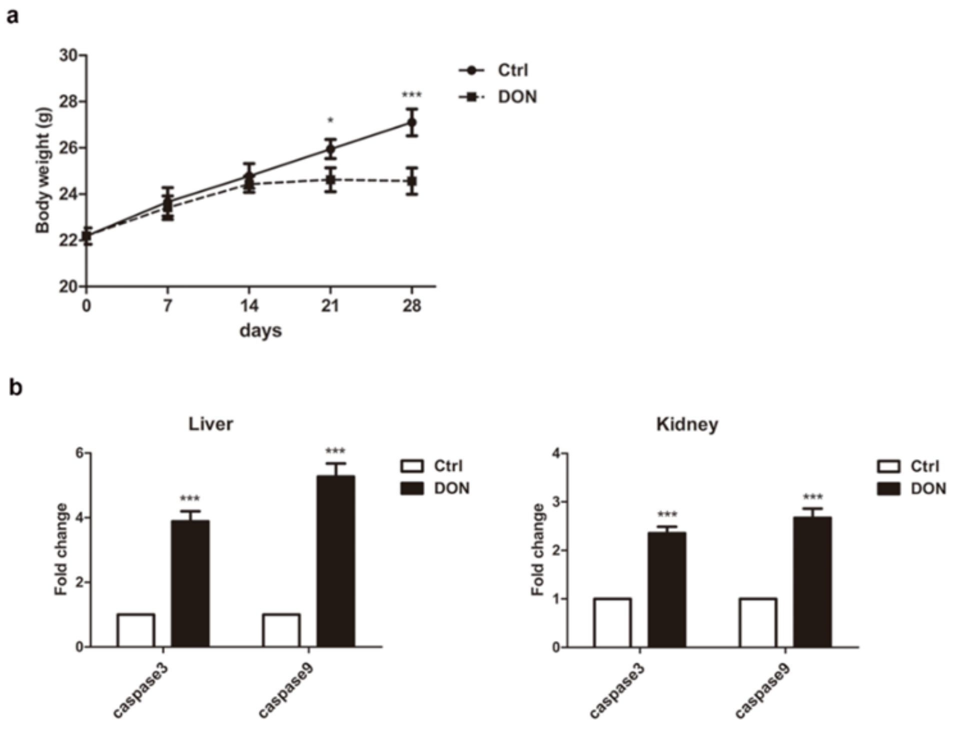

Figure S3. The effect of DON on the change in body weight, liver function, and renal function. Four-week-old SPF male BALB/c mice were randomized into two groups (n = 5/group)—the control group and DON group (3 mg/kg/day)—for four weeks. (**a**) Body weight changes. The body weights of the mice were recorded once a week. (**b**) The mRNA levels of caspase 3 and caspase 9 were determined by RT-PCR in the liver and kidney tissues of BALB/c mice exposed to DON and control groups. β-actin was used as a reference gene. The values are expressed as fold changes in mRNA levels relative to the control group. The results are representative of three independent experiments, and statistical significance is indicated by * $p < 0.05$, *** $p < 0.001$.

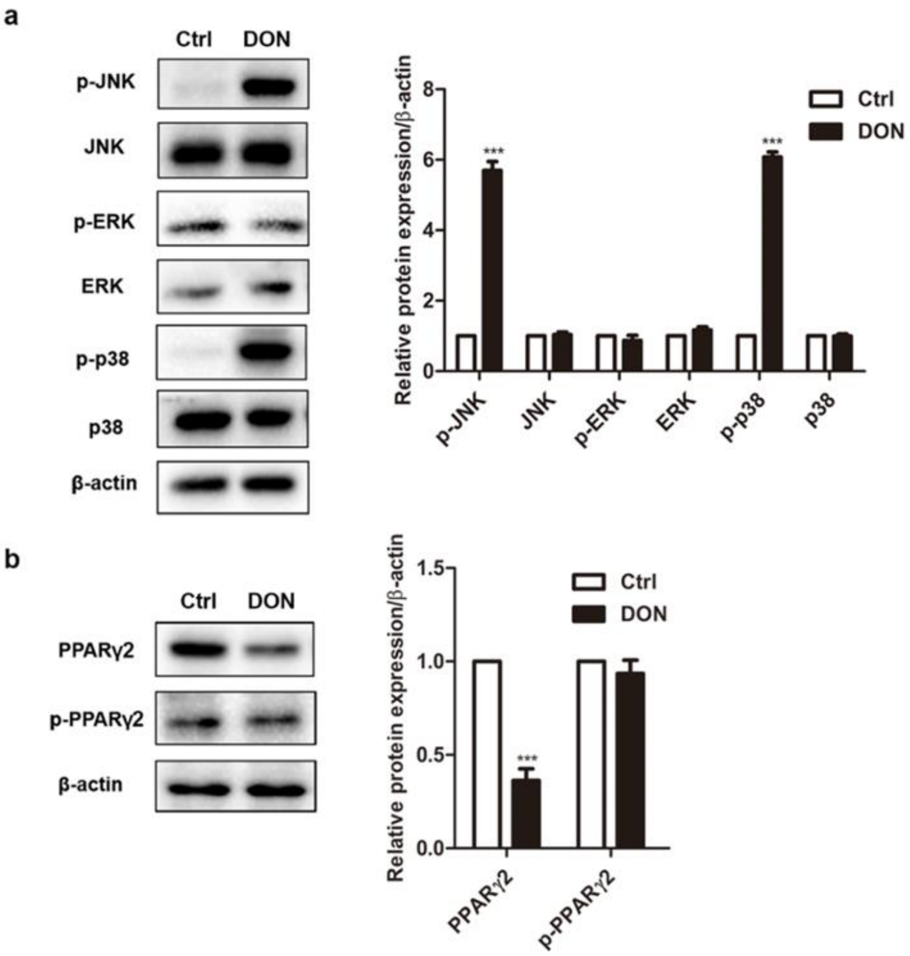

Figure S4. The effect of DON on the expression of MAPK and p-PPARγ2 in 3T3-L1 cells. (**a**) 3T3-L1 cells were differentiated and treated with or without DON (100 ng/mL) for 7 days. The expression of JNK, p-JNK, ERK, p-ERK, p38, and p-p38 was quantified by Western blotting (left). Quantification of protein levels (right). (**b**) The expression of PPARγ2 and p-PPARγ2 was quantified by Western blotting (left). Quantification of protein levels (right). The results are representative of three independent experiments, and statistical significance is indicated by *** $p < 0.001$.

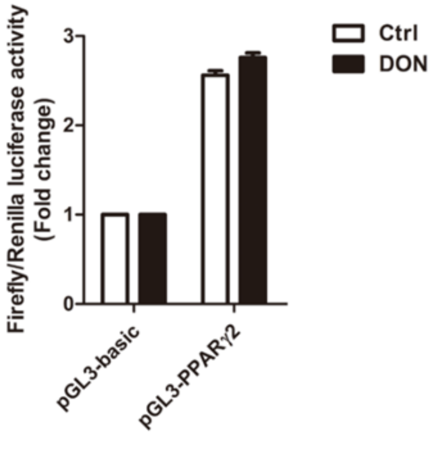

Figure S5. Analysis of the Pparγ2 promoter in HEK293T cells treated with DON. HEK293T cells were transiently transfected with the luciferase reporter plasmid pGL3-Basic, as the negative control, or the plasmids containing the promoter 1.0 kb DNA upstream of the Pparγ2 ORF. The Renilla luciferase

reporter plasmid pRL-TK was cotransfected into cells as a normalization control. The results are representative of three independent experiments.

Table S1. Primers for construction of expression vector.

Gene	Primer Sequences (5′-3′)
**ORF**	
Pparγ2-F	GGTGTCGTGAGGATTTACGTAATGGGTGAAACTCTGGGA
Pparγ2-R	GTCATCCTTGTAATCTACGTAATACAAGTCCTTGTAGATCTCC
**Promoter**	
Pparγ2-F	CGGCGAGCTCAGTCCACAAGTCACTGAA
Pparγ2-R	CGGCCTCGAGTCTATGTCTTGCAAAAGATTGG

Table S2. Primers for quantitative real-time PCR.

Genes	Primer Sequences (5′-3′)
C/ebpα-F	AGTCGGTGGACAAGAACAGC
C/ebpα-R	GTCACTGGTCAACTCCAGCA
C/ebpβ-F	ACGACTTCCTCTCCGACCTCT
C/ebpβ-R	CGAGGCTCACGTAACCGTAGT
C/ebpδ-F	ATCGACTTCAGCGCCTACAT
C/ebpδ-R	CTAGCGACAGACCCCACAC
Pparγ2-F	TTTTCAAGGGTGCCAGTTTC
Pparγ2-R	AATCCTTGGCCCTCTGAGAT
Fasn-F	TGTGAGTGGTTCAGAGGCAT
Fasn-R	TTCTGTAGTGCCAGCAAGCR
Fabp4-F	TGATGCCTTTGTGGGAACCT
Fabp4-R	GCAAAGCCCACTCCCACTT
Acaca-F	GAATCTCCTGGTGACAATGCTTATT
Acaca-R	GGTCTTGCTGAGTTGGGTTAGC
Cd36-F	ATGGGCTGTGATCGGAACTG
Cd36-R	GTCTTCCCAATAAGCATGTCTCC
caspase3-F	GAAACTCTTCATCATTCAGGCC
caspase3-R	GCGAGTGAGAATGTGCATAAAT
caspase9-F	TGTGAATATCTTCAACGGGAGC
caspase9-R	GAGTAGGACACAAGGATGTCAC
ATGL-F	CACTTTAGCTCCAAGGATGA
ATGL-R	TGGTTCAGTAGGCCATTCCT
HSL-F	CTCACAGTTACCATCTCACCTC
HSL-R	GATTTTGCCAGGCTGTTGAGTA
β-actin-F	GACAACGGCTCCGGCATGTGCAAAG
β-actin-R	TTCACGGTTGGCCTTAGGGTTCAG

Table S3. Primers for ChIP analysis.

Genes	Primer Sequences (5′-3′)
Pparγ2-F	CCAAATACGTTTATCTGGTGTTTC
Pparγ2-R	CGTTGCTACATTGTCTCGC
C/ebpα-F	TTGCGCCACGATCTCTCTC
C/ebpα-R	CTTAGAGCCCGCCTTCTCCT
